# Conservation and Diversity of Influenza A H1N1 HLA-Restricted T Cell Epitope Candidates for Epitope-Based Vaccines

**DOI:** 10.1371/journal.pone.0008754

**Published:** 2010-01-18

**Authors:** Paul ThiamJoo Tan, A. T. Heiny, Olivo Miotto, Jerome Salmon, Ernesto T. A. Marques, Francois Lemonnier, J. Thomas August

**Affiliations:** 1 Department of Pharmacology and Molecular Sciences, School of Medicine, Johns Hopkins University, Baltimore, Maryland, United States of America; 2 Department of Biochemistry, Yong Loo Lin School of Medicine, National University of Singapore, Singapore, Singapore; 3 Centre for Genomics and Global Health, Oxford University, Oxford, United Kingdom; 4 Mahidol-Oxford Research Unit, Faculty of Tropical Medicine, Mahidol University, Bangkok, Thailand; 5 Center for Vaccine Research, University of Pittsburgh, Pittsburgh, Pennsylvania, United States of America; 6 Department of Infectious Diseases and Microbiology, University of Pittsburgh, Pittsburgh, Pennsylvania, United States of America; 7 Centro de Pesquisa Aggeu Magalhaes, FIOCRUZ Department of Virology - LaViTE Recife Brazil; 8 Institut Pasteur, Unité Immunité Cellulaire Antivirale, Paris, France; New York University, United States of America

## Abstract

**Background:**

The immune-related evolution of influenza viruses is exceedingly complex and current vaccines against influenza must be reformulated for each influenza season because of the high degree of antigenic drift among circulating influenza strains. Delay in vaccine production is a serious problem in responding to a pandemic situation, such as that of the current H1N1 strain. Immune escape is generally attributed to reduced antibody recognition of the viral hemagglutinin and neuraminidase proteins whose rate of mutation is much greater than that of the internal non-structural proteins. As a possible alternative, vaccines directed at T cell epitope domains of internal influenza proteins, that are less susceptible to antigenic variation, have been investigated.

**Methodology/Principal Findings:**

HLA transgenic mouse strains expressing HLA class I A*0201, A*2402, and B*0702, and class II DRB1*1501, DRB1*0301 and DRB1*0401 were immunized with 196 influenza H1N1 peptides that contained residues of highly conserved proteome sequences of the human H1N1, H3N2, H1N2, H5N1, and avian influenza A strains. Fifty-four (54) peptides that elicited 63 HLA-restricted peptide-specific T cell epitope responses were identified by IFN-γ ELISpot assay. The 54 peptides were compared to the 2007–2009 human H1N1 sequences for selection of sequences in the design of a new candidate H1N1 vaccine, specifically targeted to highly-conserved HLA-restricted T cell epitopes.

**Conclusions/Significance:**

Seventeen (17) T cell epitopes in PB1, PB2, and M1 were selected as vaccine targets based on sequence conservation over the past 30 years, high functional avidity, non-identity to human peptides, clustered localization, and promiscuity to multiple HLA alleles. These candidate vaccine antigen sequences may be applicable to any avian or human influenza A virus.

## Introduction

Influenza A viruses are major pathogens of avian origin with global spread and rapid mutational change, some of which also infect humans and other mammals. Of particular concern are the several ways a human influenza pandemic could emerge. One is through the occurrence of a novel and highly pathogenic zoonotic strain capable of infecting humans, such as the H5N1 avian pathogen. Another possibility is through mutation from a mild to a more pathogenic human transmissible strain, such as mutation to the current H1N1 strain. The most threatening is mutations giving rise to a new highly transmissible-and-pathogenic human strain, as occurred with the original 1918 Spanish influenza. In any event, history teaches us that a vaccine to prevent a new influenza A pandemic must be effective against all future forms of the virus.

Influenza A viruses are single stranded, negative-sense RNA viruses belonging to the family *Orthomyxoviridae*. The genome is composed of 8 RNA strands of about 13,500 bases, encoding at least ten viral proteins. The viral envelope is a lipid bilayer, consisting of the interior matrix protein 1 (M1) and three exterior transmembrane proteins: hemagglutinin (HA), neuraminidase (NA), and matrix protein 2 (M2). The viral core contains viral ribonucleoprotein complex particles, consisting of viral RNA, nucleoprotein (NP), and three polymerase proteins (PB1, PB2, and PA). Mutation in the viral RNA genome occurs by two mechanisms, known as antigenic drift and antigenic shift. Antigenic drift is the frequent occurrence of point mutations resulting from defects in RNA replication mechanisms, while antigenic shift is less frequent, involving re-assortment of the RNA segments arising from exchanges between different strains in host cells infected by multiple viruses.

Protection by current human influenza vaccines is achieved by use of inactivated or attenuated forms of the corresponding pathogen and appears to require the function of neutralizing antibodies against the external HA and NA glycoproteins. However, these glycoproteins mutate rapidly through antigenic drift and current vaccines become ineffective as mutational differences accumulate in the circulating strains. In order to overcome the antigenic variability of influenza external glycoproteins, new vaccine strategies are increasingly directed at conserved regions of the viral proteins for production of T cell epitope-based vaccines. The goal is to identify conserved sequences that function as epitopes recognized by human leukocyte antigen (HLA) molecules for presentation to CD8^+^ and CD4^+^ T cells that provide immunity against all influenza A virus subtypes and obviate the need for yearly vaccine update. Several animal model studies taking this approach have reported T cell responses that reduce morbidity and promote recovery in mouse models of influenza challenge [1]–[4]. Both CD8^+^ and CD4^+^ T cell responses are required; CD8^+^ T cells to kill infected cells [5], [6] and CD4^+^ T cells for the development of an effective immune response and immune memory [7]–[9]. A complication of cellular immunity is that T cell responses are dependent upon antigen presentation by highly polymorphic HLA molecules that vary greatly among human populations. However, the limited population coverage of some HLA alleles may be alleviated by focusing on T cell epitopes recognized by HLA supertypes that bind largely overlapping peptide repertoires on the basis of the specificity for the main anchor positions of the presented peptides [10], [11].

We previously reported a detailed study of evolutionarily conserved sequences of all human and avian influenza A viruses that were recorded over the past 30 years (36,343 sequences) [12]. Fifty-four (54) sequences, ranging from 9 to 58 amino acids (aa) of the PB2, PB1, PA, NP, and M1 sequences were conserved in at least 80%, and in most cases 95–100% of all recorded human H1N1, H3N2, H1N2, and H5N1, and avian subtypes. These sequences have remained evolutionarily stable for all recorded human and avian influenza A viruses during the past decades, and are thus prime candidates for the development of T cell epitope-based vaccines against multiple influenza strains. However, the function of these conserved sequences as HLA-restricted T cell epitopes and the incidence of variant sequences in association with the conserved sequences were not known. Herein, we have focused on the identification and characterization of peptides of influenza virus A/New York/348/2003 (H1N1) that contain conserved sequences and elicit HLA-restricted T cell responses. HLA transgenic mice (HLA-A2, -A24, -B7, -DR2, -DR3, and -DR4) were immunized with the selected peptides. The peptides that elicited T cell activation by IFN-γ ELISpot assay and thus contained T cell epitopes were selected and analyzed for properties relevant to vaccine development, including evolutionary conservation and diversity, and correspondence to the 2007–2009 human H1N1 sequences.

## Materials and Methods

### Ethics Statement

Mice were maintained in a pathogen-free facility at the Johns Hopkins University according to IACUC guidelines.

### Influenza Peptides

Peptide arrays of PB2 (BEI Cat.: NR-2616), PB1 (NR-2617), PA (NR-2618), NP (NR-2611), and M1 (NR-2613) of influenza virus A/New York/348/2003 (H1N1) were obtained through the NIH Biodefense and Emerging Infections Research Resources Repository, NIAID, NIH (BEI). A total of 196 peptides (all 17 aa long except PB2_268–283_ and _393–408_) were selected to fully cover all highly conserved sequences under study. Where these sequences spanned two or more 17 aa peptides, the consecutive peptides were overlapped by 11 aa (Figure S1). Two immunization peptide pools for immunization were formed: one composed of 84 PB2 and 13 M1 peptides (Table S1), and the second composed of 48 PB1, 23 PA, and 28 NP peptides (Table S2). Each of the 196 peptides was dissolved in 100% DMSO and constituted to 20% with sterile filtered water. The final concentration of each peptide was 2 µg/µl. The dissolved peptides were stored at −20°C.

### HLA Transgenic Mice

Six different strains of HLA transgenic mice were used to cover HLA alleles of class I and class II supertypes. These six alleles were selected based on their prevalence in the Caucasian population and the availability of HLA transgenic mouse strains in our laboratory. The HLA class I supertypes studied were HLA-A2 (A*0201) mice expressing a chimeric heavy chain with murine α3 domain and human β2m. Both H-2D^b^ and murine β2m genes were disrupted by homologous recombination [13], HLA-A24 (A*2402) mice express a chimeric heavy chain and human β2m; the H-2K^b^, H-2D^b^, and murine β2m genes were disrupted by homologous recombination (Lemonnier *et al*., unpublished), HLA-B7 (B*0702) mice express a chimeric heavy chain with the HLA-B*0702 α1 and α2 domains and the H-2K^d^ murine α3 domain [14]. The H-2K^b^ and H-2D^b^ genes in HLA-B7 mice were inactivated by homologous recombination. The HLA-A2 and -B7 transgenic mice were kind gifts from Steve Pascolo (Institut Pasteur, France) and Pierre-Simon Rohrlich (Institut Pasteur, France), respectively.

The HLA class II supertypes studied herein were DR2 (DRB1*1501), DR3 (DRB1*0301), and DR4 (DRB1*0401) where HLA-DR2, -DR3, and -DR4 transgenic mice were kind gifts from Lars Fugger (Weatherall Institute of Molecular Medicine, Oxford, UK) and Arthur Vanderbark (Oregon Health and Science University, Portland), Chella S. David (Mayo Clinic, Rochester), and Grete Sønderstrup (Stanford University School of Medicine), respectively. The peptide-binding domain of HLA-DR2 transgenic mice is encoded by human sequences, while the membrane proximal portion containing the CD4-binding domain is encoded by mouse sequences (DRA1*0101: I-Eα and DRB1*1501: I-Eβ) [15]. HLA-DR3 transgenic mice express HLA-DRA*0101 and -DRB1*0301 [16]. HLA-DR4 transgenic mice express HLA-DRA*0101, -DRB1*0401, and human CD4 [17]. The derivation and validation of the above transgenic mice has been described in the cited publications.

### Immunization

Mice were immunized with the selected 196 peptides in 2 pools by use of a protocol which had been validated for T cell studies [18] and adapted for these transgenic mice studies. Peptides were pooled in matrixes as described [19] and injected in groups of 9 mice of each transgenic strain: two for matrix array screening, two for identifying individual peptides, four for characterizing apparent functional avidity of T cells to positive peptides at three titration points: 10, 1, and 0.1 µg/ml peptide concentrations, and one as a control (adjuvant alone). Mice were injected subcutaneously at the base of tail with 100 µl of the immunization peptide pool in TiterMax® Gold adjuvant (TiterMax, Norcross, GA) (1∶1). The amount of each peptide injected was 1 µg/mouse. After two weeks, spleens were harvested for IFN-γ ELISpot assay.

### IFN-γ ELISpot Assay

Harvested spleens from immunized transgenic mice were selectively depleted of CD8^+^ or CD4^+^ T cells by use of anti-CD8 or anti-CD4 antibody-coated immunomagnetic beads with LD columns (Miltenyi Biotec, Auburn, CA) according to the manufacturer's protocol. The resulting CD8^+^ or CD4^+^ depleted splenocytes were tested individually by IFN-γ ELISpot assays against the 196 influenza peptides arranged in two 10×10 matrix arrays, resulting in 40 peptide pools, where each peptide was present in two different pools, as described [19]. Peptides identified as immunogenic in the matrix array screen were retested individually in a confirmatory assay and a peptide titration assay. Thus, each ELISpot positive response was detected three times: by matrix array screening, individually by confirmatory assay, and by peptide titration.

The ELISpot assays were performed using mouse IFN-γ ELISpot sets from BD Biosciences (San Jose, CA) according to the manufacturer's protocol. Briefly, the ELISpot plates were coated with anti-IFN-γ at 5 µg/ml and incubated at 4°C overnight. The plates were blocked with RPMI 1640 containing 10% heat-inactivated fetal calf serum, 2 mM L-glutamine, 100 µg of streptomycin/ml, and 100 U of penicillin for 2 h at room temperature, and either CD8^+^- or CD4^+^-depleted splenocytes (0.5−1.0×10^6^ cells/well) were then added for assays of class II and I T cell epitopes, respectively. The cells were cultured at 37°C in 5% CO_2_ in the presence of peptide pools (final concentration of each peptide was 10 µg/ml) or individual peptides at final concentrations of 10 µg/ml, 1 µg/ml, and 0.1 µg/ml. Wells with medium alone served as background; Concanavalin A (2.5 µg/ml; Sigma-Alrich, St. Louis, MO) was used as a polyclonal stimulator; and known HLA-restricted peptides from Dengue serotype 3 were included in each assay as positive controls. After 16 h of culture, the plates were washed and incubated with biotinylated anti-IFN-γ for 2 h at room temperature, followed by HRP-conjugated streptavidin for 1 h at room temperature. Reactions were developed with AEC substrate (Calbiochem-Novabiochem, San Diego, CA). Final enumeration of IFN-γ spot-forming cells (SFC) was performed using the Immunospot Series 3B Analyzer ELISPOT reader (Cellular Technologies, Shaker Heights, OH) with aid of the Immunospot software version 3.0 (Cellular Technologies), indicating the number of SFC/10^6^ cells. The results were considered positive if the number of SFC subtracted by those in the background (culture with medium alone) was above 10 and the number of SFC was higher than the background plus two standard deviations. The results shown are SFC minus background, which was consistently found to be less than 15 spots/10^6^ cells throughout the experiments.

### Experimentally Identified Human T Cell Epitopes in the Highly Conserved Sequences of Influenza A Viruses

Published influenza T cell epitopes within the highly conserved sequences were identified by use of the Immune Epitope Database and Analysis Resource (IEDB, http://www.immuneepitope.org/) [20]. The published epitope sequences were derived from various T cell assays that included T cell proliferation, IFN-γ ELISpot, HLA tetramer staining, and ^51^Cr release assays. Only epitope data from unique sequences and containing HLA restriction information were included.

### Determination of Human Self-Peptide in Influenza Peptides

The 196 influenza 17 aa peptides were compared using the blastp program against the non-redundant protein sequences database restricted to human (taxid:9606) at NCBI (http://www.ncbi.nlm.nih.gov/BLAST/) to detect the presence of fragments identical to human peptides. As the default search parameters were not suitable to probe for short peptide sequences of length 30 or less, the following parameters were used: word size of 2, expectation value of 30,000, matrix was PAM30, low complexity filter was disabled, and composition-based statistics was set to ‘no adjustment’. We disregarded search results containing predicted sequences and human peptides with fewer than six contiguous identical residues as the probability of matching five or less residues is high and non-significant.

### Conservation of Influenza A (H1N1) T Cell Epitopes

The dataset and methodology for identification of highly conserved influenza protein sequences among pathogenic influenza strains for the past 30 years had been described by Heiny *et al.* [12]. Briefly as reported, 3763 NP, 3781 M1, 3111 PA, 3175 PB1, and 3144 PB2 sequences were extracted from the NCBI GenBank and GenPept databases (as of September 2006) and multiple sequence alignments of the individual proteins were performed. The Antigenic Variability Analyzer (AVANA) tool [21] was used to identify the most frequent 17 aa sequence present in at least 80% of all the recorded viruses.

The 2007–2009 human H1N1 sequences were compared with the T cell epitopes of A/New York/348/2003 (H1N1), by aligning the protein sequence records of human H1N1 M1, PB1, and PB2 retrieved from the NCBI Influenza Virus Sequence Database (http://www.ncbi.nlm.nih.gov/genomes/FLU/FLU.html, as of Jun 17, 2009) and application of the AVANA tool to identify the most frequent sequence and its variants for each year.

## Results

### HLA-Restricted Epitopes of Highly Conserved Human and Avian Influenza A Virus Sequences

The previously described 54 highly conserved influenza A sequences of 9 or more contiguous aa of the recorded human and avian influenza strains were represented by a total of 956 aa of the M1, NP, PA, PB1, and PB2 proteins [12]. The majority of the conserved sequences, 650 aa, were in the PB1 and PB2 proteins; there were no conserved sequence in NA, M2, NS1, and NS2. A total of 196 peptides (BEI) of the A/New York/348/2003 (H1N1) M1, NP, PA, PB1, and PB2 proteins were selected based on the presence of the conserved sequences. The immunogenicity of these 196 conserved influenza peptides was studied by immunizing HLA-A2, -A24, -B7, -DR2, -DR3, and -DR4 transgenic mice. Organization of the 54 conserved sequences in the BEI 17 aa peptides depended on their length and position. Conserved sequences that spanned adjacent 17 aa peptides were repeated up to a maximum of 11 aa because of overlapping peptide synthesis (Tables S1 & S2, Figure S1). Peptides with conserved sequences of less than 17 aa contained mixtures of conserved and non-conserved aa. Thirty-two (32) short conserved sequences (9 to 16 aa) were present in various lengths with adjacent non-conserved aa. Conserved sequences of greater length (22 sequences of 17 to 57 aa) were present as complete (65 of the 196 peptides) or partial sequences in the overlapping peptides. The longest conserved sequence was PB1_518–575_ which was included as part of a cluster of completely conserved aa of 7 overlapping peptides.

**Figure 1:**
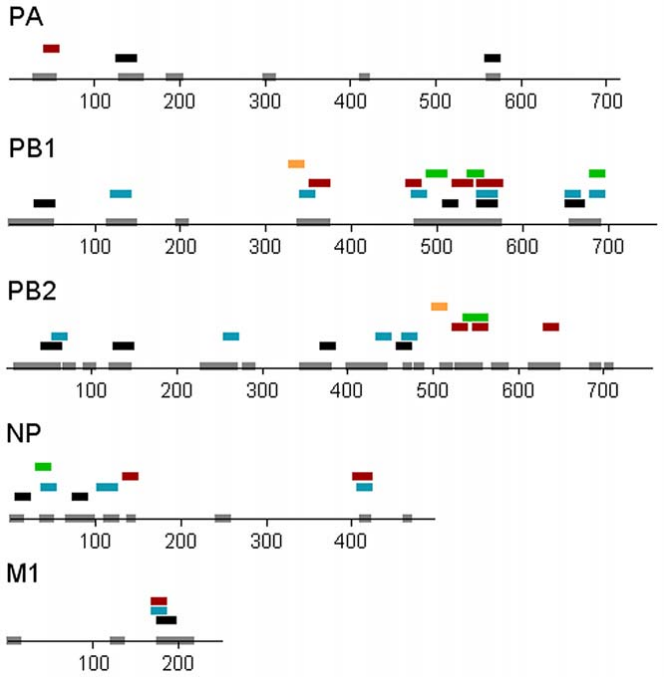
Localization of HLA-restricted T cell epitopes of conserved sequences of influenza polymerases, NP, and M1 proteins. Numbers represent aa positions. Highly conserved aa are shown as grey boxes. T cell epitopes were restricted by HLA-DR4 (black boxes), -DR3 (blue boxes), -DR2 (brown boxes), -A24 (green boxes), and -B7 (orange boxes).

Immunization of the HLA transgenic mice with the 196 H1N1 peptides was carried out with 2 pools of about 100 peptides each, with groups of 9 mice of each transgenic strain. Interferon-γ (IFN-γ) ELISpot assays for HLA-restricted class I and class II responses were performed with splenocytes of the immunized mice that were depleted of CD4^+^ and CD8^+^ T cells, respectively, to identify the responding T cell subset. The initial assays contained matrix arrays of peptide pools followed by validation assays with individual peptides [19]. Of the 196 peptides, 54 contained T cell epitopes that elicited 63 ELISpot responses (8 A24, 2 B7, 16 DR2, 17 DR3, and 20 DR4) (Table 1). None of the 196 peptides tested induced T cell responses in mice expressing the HLA-A2 allele. Forty-seven (47) of the 54 epitopes were restricted by one HLA allele; eight by class I HLA-A24 and -B7, and 39 by class II HLA-DR2, -DR3, and -DR4. The remaining 7 peptides contained epitopes that were presented by at least two HLA alleles of distinct supertypes i.e. they contained multiple or promiscuous T cell epitopes. Epitopes of PB1_680–696_ and PB2_548–564_ were presented by both HLA class I and II alleles. Sixteen (16) pairs of consecutive peptides were restricted by the same HLA allele, possibly because there were identical epitopes in the overlapping 11 aa sequence shared by the 2 adjacent peptides. Clusters of 2 or more T cell epitopes with at least 16 conserved aa were M1_175–197_, PB1_120–142, 340–374, 489–576_, and PB2_42–64, 126–146_ (Table 1, Figure 1).

**Table 1:** HLA-A24, -B7, -DR2, -DR3, and -DR4 restriction of 54 peptides of influenza proteins M1, NP, PA, PB1, and PB2 that contain conserved sequences of 9 or more amino acids.

Protein	ELISpot positive 17 aa peptide*	A24#	B7	DR2	DR3	DR4
M1	169 TNPLIR**HENRMVLASTT** 185	-	-	56±5 (0.1)	120±4 (0.1)	-
	175 **HENRMVLASTTAKAMEQ** 191	-	-	-	-	165±1 (0.1)
	181 **LASTTAKAMEQMAGSSE** 197	-	-	-	-	115±21 (1)
NP	7 **KRSYEQMET**DGERQNAT 23	-	-	-	-	52±29 (0.1)
	31 RMIG**GIGRFYIQMCTEL** 47	45±5 (0.1)	-	-	-	-
	37 **GRFYIQMCTELKL**NDYE 53	-	-	-	66±7 (1)	-
	73 **ERRN**K**YLEEHPSAGKDP** 89	-	-	-	-	121±1 (0.1)
	103 KWVRELV**LYDKEEIRRI** 119	-	-	-	614±21 (0.1)	-
	109 V**LYDKEEIRRIWRQANN** 125	-	-	-	501±42 (0.1)	-
	133 LTHI**MIWHSNLND**TTYQ 149	-	-	238±59 (0.1)	-	-
	402 SAGQIST**QPTFSVQRNL** 418	-	-	207±3 (0.1)	-	-
	408 T**QPTFSVQRNLPF**DKTT 424	-	-	110±14 (1)	41±2 (10)	-
PA	42 **LEVCFMYSDFHFI**NEQG 58	-	-	64±11 (1)	-	-
	126 EVHI**YYLEKANKIKSE**K 142	-	-	-	-	37±11 (0.1)
	132 **LEKANKIKSE**K**THIHIF** 148	-	-	-	-	41±10 (0.1)
	558 **SRPMFLYVRTNGTSK**IK 574	-	-	-	-	114±24 (0.1)
PB1	31 **SHGTGTGYTMDTVNRTH** 47	-	-	-	-	106±1 (0.1)
	37 **GYTMDTVNRTHQYSE**RG 53	-	-	-	-	125±11 (0.1)
	120 **DKLTQGRQTYDWTLNRN** 136	-	-	-	142±6 (0.1)	-
	126 **RQTYDWTLNRNQPAATA** 142	-	-	-	78±0 (0.1)	-
	328 NQPEWFRNI**LSIAPIMF** 344	-	60±8 (10)	-	-	-
	340 **APIMFSNKMARLGKGYM** 356	-	-	-	175±0 (0.1)	-
	352 **GKGYMFESK**S**MKLRTQI** 368	-	-	52±2 (1)	-	-
	358 **ESK**S**MKLRTQIPAEMLA** 374	-	-	84±20 (0.1)	-	-
	465 RFYRTCKLL**GINMSKKK** 481	-	-	231±73 (1)	-	-
	471 KLL**GINMSKKKSYIN**R**T** 487	-	-	-	116+10 (0.1)	-
	489 **TFEFTSFFYRYGFVANF** 505	213±9 (0.1)	-	-	-	-
	495 **FFYRYGFVANFSMELPS** 511	210±25 (0.1)	-	-	-	-
	507 **MELPSFGVSG**V**NESADM** 523	-	-	-	-	274±15 (0.1)
	519 **ESADMSIGVTVIKNNMI** 535	-	-	75±10 (0.1)	-	-
	525 **IGVTVIKNNMINNDLGP** 541	-	-	159±53 (0.1)	-	-
	537 **NDLGPATAQMALQLFIK** 553	92±2 (1)	-	-	-	-
	548 **LQLFIKDYRYTYRCHRG** 564	-	-	61±2 (1)	230+23 (0.1)	97±30 (0.1)
	554 **DYRYTYRCHRGDTQIQT** 570	-	-	109±13 (1)	166±22 (0.1)	76±2 (0.1)
	560 **RCHRGDTQIQTRRSFE**I 576	-	-	194±47 (0.1)	-	-
	650 GPAKN**MEYDAVATTHSW** 666	-	-	-	142±45 (0.1)	41±9 (0.1)
	656 **EYDAVATTHSW**V**PKRNR** 672	-	-	-	-	59±2 (0.1)
	680 **RGILEDEQMYQ**RCCNLF 696	78±4 (0.1)	-	-	181±10 (0.1)	-
PB2	42 **NP**S**LRMKWMMAMKYPIT** 58	-	-	-	-	166±3 (0.1)
	48 **KWMMAMKYPITADKRI**T 64	-	-	-	-	161±18 (0.1)
	54 **KYPITADKRI**TEMI**PER** 70	-	-	-	499±4 (0.1)	-
	126 **KHGTFGPVHFRNQVKIR** 142	-	-	-	-	316±20 (0.1)
	132 **PVHFRNQVKIRRRVD**IN 148	-	-	-	-	311±37 (0.1)
	256 **DQSLIIAARNIVRRA**AV 272	-	-	-	169±12 (0.1)	-
	369 **RATAILRKATRR**LIQLI 385	-	-	-	-	54±2 (0.1)
	434 **LLRHFQKDAKVLF**LNWG 450	-	-	-	444±14 (0.1)	-
	458 MGMIGILP**DMTPSTEMS** 474	-	-	-	-	238±5 (0.1)
	464 LP**DMTPSTEMS**MRGVRV 480	-	-	-	324±28 (0.1)	-
	500 RFLRVRDQR**GNVLLSPE** 516	-	184±3 (0.1)	-	-	-
	524 TEK**LTITYSSSMMWEIN** 540	-	-	151±67 (0.1)	-	-
	536 **MWEINGPESVL**I**NTYQW** 552	289±16 (0.1)	-	-	-	-
	542 **PESVL**I**NTYQWIIRNWE** 558	226±5 (0.1)	-	-	-	-
	548 **NTYQWIIRNWE**TVKIQW 564	322±44 (0.1)	-	96±9 (0.1)	-	-
	630 **RMQFSSLTVNVRGSGMR** 646	-	-	104±16 (0.1)	-	-
	ELISpot responses	8	2	16	17	20

*Conserved aa are boldface. Consecutive peptides overlapping by 11 aa are aligned.

#Numbers are representative average IFN-γ spots forming cells per million splenocytes of individual transgenic mice that were positive at 10 µg/ml of peptide concentration. Number (10, 1 or 0.1) in parenthesis represents the lowest concentration of peptide (µg/ml) giving positive ELISpot response in peptide titration.

-represents no positive IFN-γ ELISpot response.

The apparent functional avidity of T cells to each of the 54 peptides was titrated at three peptide concentrations of 10, 1 and 0.1 µg/ml in IFN-γ ELISpot assays. Of the 63 positive ELISpot responses, including the responses of peptides restricted by multiple HLA alleles, 52 activated IFN-γ secretion at each of the three concentrations used in the ELISpot assay, 9 elicited at concentrations of 10 and 1 µg/ml, and 2 peptides (NP_408–424_ and PB1_328–344_) elicited solely at the highest peptide concentration (Table 1).

### Presence of Reported Human T Cell Epitopes in the Conserved Sequences of Influenza A

The conserved peptides of this study were compared with reported T cell epitope sequences of humans infected with influenza A viruses extracted from the IEDB [20]. Twenty-one (21) of about 800 reported human T cell epitopes of PB2, PB1, PA, NP, and M1 were found to contain sequences of 9 or more conserved amino acids of all recorded 1977–2006 influenza A viruses (Table 2). These HLA-restricted T cell epitopes were mainly from H1N1, H3N2, and H5N1 infections and included sequences restricted by a broad range of HLA class I and II alleles, including many alleles not covered by the transgenic mice of this study. For example, the same T cell epitope “RMVLASTTAK” in M1_178–187_ was reported to be restricted by HLA-A3 and -A11 [22], [23]. Clusters of overlapping epitopes were also observed within the conserved sequences, for example, M1_123–137_ had three overlapping epitopes (_123_ ALASCMGLIY _132_ was restricted by A1; _125_ ASCMGLIY _132_ by B35; and _129_ GLIYNRMGA _137_ by A2) [22], [24]. Thus, the highly conserved sequences contained common epitopes shared by pathogenic influenza strains and could be restricted by a broad range of HLA alleles.

**Table 2:** Presence of reported human influenza A T cell epitopes in 21 highly conserved sequences of A/New York/348/2003 (H1N1).

Highly conserved 17 aa peptide*	HLA allele this work#	Published HLA alleles	Influenza strain
M1 1 **M** ***SLLTEVETYV*** **LSI**VPS 17	-	A2	A/Puerto Rico/8/34 (H1N1)
M1 121 A**G** ***ALASCMGLIYNRMG*** *A* 137	-	A1, A2, B35, DRB1*0404	A/Vietnam/1203/2004 (H5N1), Influenza A (H3N2)
M1 169 TNPL*IR* ***HENRMVL*** **ASTT** 185	DR2, DR3	B39, DR2, DRB1*0103, DRB1*1101, DRB1*0701, DRB5*0101	A/Vietnam/1203/2004 (H5N1), Influenza A
M1 175 **HEN** ***RMVLASTTAK*** **AMEQ** 191	DR4	A3, A11, DRB1*0701	A/Puerto Rico/8/34 (H1N1), A/Vietnam/1203/2004 (H5N1)
NP 61 LTIE*R* ***MVLSAFDER*** **RN**K 77	-	A3	Influenza A
NP 67 **VLSAFDERRN**K**YLEEHP** 83	-	DRB1*0101	A/Vietnam/1203/2004 (H5N1)
NP 73 **ERRN**K**YLEEHPSAGKDP** 89	DR4	DR1, DRB1*0101	A/NT/60/68 (H3N2), A/Vietnam/1203/2004 (H5N1)
NP 91 ***KTGGPIY*** *KR*VDGKWVRE 107	DR3	A68	A/Texas/1/77 (H3N2)
NP 109 V**LYDKEEIRRIWRQANN** 125	DR3	DRB1*1101	A/Vietnam/1203/2004 (H5N1)
NP 402 SAGQIST**QPTFSVQRNL** 418	DR2	DRB1*0101, DRB1*0404	A/Vietnam/1203/2004 (H5N1)
PA 42 **LEVC** ***FMYSDFHFI***NEQG 58	DR2	A2	A/Puerto Rico/8/34 (H1N1)
PB1 1 **MDVNPT** ***LLFLKVP*** *A* **QNA** 17	-	A2	Influenza A
PB1 37 **GYTM** ***DTVNRTHQY*** **SE**RG 53	DR4	A26	Influenza A
PB1 346 **N** ***KMARLGKGYMF*** **ESK**S**M** 362	-	B62, B27	Influenza A
PB1 352 **GKGYM** ***FESK*** *S* ***MKL*** **RTQI** 368	DR2	B44	Influenza A
PB1 489 ***TFEFTSFFY*** **RYGFVANF** 505	A24	A1, B44	Influenza A
PB1 501 ***FVANFSMELPSFGV*** **SG**V 517	-	A2	Influenza A
PB1 537 **NDL** ***GPATAQMAL*** **QLFIK** 553	A24	B7	Influenza A
PB1 560 **RCHRGD** ***TQIQTRRSF*** **E**I 576	DR2	B62	Influenza A
PB1 566 **TQIQT** ***RRSFEI*** *KKL*WDQ 582	-	B27	Influenza A (H3N2)
PB2 48 **K** ***WMMAMKYPI*** **TADKRI**T 64	DR4	A2	A/Puerto Rico/8/34 (H1N1)

*Conserved aa are boldface. Published HLA epitopes were extracted from the IEDB. HLA class I epitopes are italicized. HLA class II epitopes longer than 17 aa are represented only by the corresponding residues in the 17 aa peptides of A/New York/348/2003 (H1N1).

# - represents no positive IFN-γ ELISpot response.

### Analysis of the Presence of Human Peptide Sequences in Influenza Peptides

Each of the 196 influenza 17 aa peptides used in this study was compared with human proteome sequences to investigate the possibility of identity to human self-antigens that could trigger an autoimmune response to immunization. Specifically, we screened for exactly identical sequences of at least 8 continuous aa, the minimum binding peptide length for MHC class I [25]. Many of the conserved sequences of the influenza peptides contained sequences of 6 aa found in human proteins such as voltage-gated sodium channel, dystrophin etc. The longest influenza A sequence with an identical human counterpart was 7 aa of PA_131–137_ but none contained sequences of 8 or more aa identical to the human proteome (Table 3).

**Table 3:** Determination of human self-peptides in representative influenza 17aa peptides.

Viral peptide*	Human peptide	Human protein name	GenPept ID
M1 169 TNPLIR**HENR** ***MVLAST*** **T** 185	26 MVLAST 31	Ring finger protein 220	NP_060620
M1 175 **HENRMVLAST** ***TAKAME*** **Q** 191	140 TAKAME 145	Mediator of cell motility 1	NP_057039
M1 181 **LASTTAKAM** ***EQMAGS*** **SE** 197	1387 EQMAGS 1392	MYST histone acetyltransferase 3	NP_001092882
NP 7 ***KRSYEQ*** **MET**DGERQNAT 23	582 KRSYEQ 587	Metastasis associated protein	NP_004680
NP 103 KWVRELV**LYDK** ***EEIRRI*** 119	121 EEIRRI 126	Annexin IV	NP_001144
NP 402 SAGQIST**Q** ***PTFSVQ*** **RNL** 418	80 PTFSVQ 85	Mucin 6, gastric	NP_005952
NP 408 T***QPTFSV*** **QRNLPF**DKTT 424	1805 QPTFSV 1810	Chromodomain helicase DNA binding protein 9	NP_079410
PA 126 EVHI**Y** ***YLEKANK*** **IKSE**K 142+	1266 YLEKANK 1272	Dystrophin Dp427c isoform	NP_000100
	1274 YLEKANK 1280	Dystrophin Dp427m isoform	NP_003997
	1151 YLEKANK 1157	Dystrophin Dp427l isoform	NP_003998
	1270 YLEKANK 1276	Dystrophin Dp427pl isoform	NP_004000
PB1 31 **SHGTGT** ***GYTMDT*** **VNRTH** 47	3151 GYTMDT 3156	Polydom	NP_699197
PB1 31 **SHGTG** ***TGYTMD*** **TVNRTH** 47	2141 TGYTMD 2146	Multiple EGF-like-domains 8	NP_001401
PB1 471 KLL**GIN** ***MSKKKS*** **YIN**R**T** 487	609 MSKKKS 614	Suppressor variegation 4–20 homolog 1 isoform 1	NP_060105
PB1 489 **TFEFT** ***SFFYRY*** **GFVANF** 505	561 SFFYRY 566	Phosphatidylinositol glycan anchor biosynthesis	NP_036459
PB1 537 **NDLG** ***PATAQM*** **ALQLFIK** 553	919 PATAQM 924	Rho GTPase-activating protein	NP_055530
PB1 548 **LQLFIK** ***DYRYTY*** **RCHRG** 564	231 DYRYTY 236	Syntaxin binding protein 5 isoform a	NP_640337
PB2 256 **DQSLIIA** ***ARNIVR*** **RA**AV 272	725 ARNIVR 730	Akt substrate AS250	NP_065076
PB2 256 **DQSLI** ***IAARNI*** **VRRA**AV 272	1301 IAARNI 1306	ATP-binding cassette, sub-family A, member 6	NP_525023
PB2 458 MGMIGILP***DMTPST*** **EMS** 474	1964 DMTPST 1969	Voltage-gated sodium channel Type II, isoform 1	NP_066287
PB2 458 MGMIGILP***DMTPST*** **EMS** 474	1964 DMTPST 1969	Voltage-gated sodium channel Type II, isoform 2	NP_001035233

*Conserved aa are boldface.

+ PA_131–137_ shared 7 aa identity with human Dystrophin Dp427 isoform proteins.

### Variants of the Conserved T Cell Epitope Sequences

The 54 HLA-restricted T cell epitopes of A/New York/348/2003 (H1N1) strain were analyzed by the Antigenic Variability Analyzer (AVANA) tool [21] for identification of (a) the consensus sequence (most frequent sequence) in the context of influenza A conserved sequences over the past 30 years, and (b) variants and percentage conservation of 2007–2009 human H1N1 strains as compared to the 2003 H1N1 strain. Based on their conservation and diversity, the 54 T cell epitopes formed three groups:

1. Seventeen (17) T cell epitope sequences of the 2003 strain (11 PB1, 4 PB2, and 2 M1) were conserved in at least 88% of all recorded human and avian influenza strains (Table 4). In particular, PB1_489–505_ was 100% conserved in all H1N1 viruses. Several variant sequences within this group were recorded, but these were mostly single conservative amino acid substitutions representing a small fraction (less than 5%) of all the recorded 1977–2006 virus sequences. The major change in 2009 was the apparent complete replacement of 2 previous consensus sequences by variant sequences, each with 1 mutated aa (PB2_132–148, 630–646_).
2. A group of 9 PB1 and PB2 T cell epitopes of the A/New York/348/2003 H1N1 strain was variants of the 1977–2006 total recorded influenza A virus population at a single mutated aa position (Table 5). These variant A/New York/348/2003 strain sequences represented less than 15% of the consensus sequences of the entire 1977–2006 avian and human virus population. One of these, PB1_507–523_, became the H1N1 consensus sequence of 2007–2009. For the others, a single aa modification to the BEI peptide would result in 96–100% representation in the 2009 human H1N1 population.
3. The remaining 28 peptides were each represented in the dataset by 2 to 7 variant sequences with multiple mutations (Table S3). The A/New York/348/2003 sequences were the predominant form in only 13 of the 28 peptides and at reduced representations of 6 to 72% of the recorded viruses. As the variant forms contained a mixture of conserved sequences and variable amino acids, it is not possible to predict the immunogenicity of the variant sequences represented in nature and their use as vaccine sequences. These data demonstrated that when T cell epitopes contain mixtures of conserved and non-conserved aa, the occurrences of mutated sequences in a subsequent influenza A strain are greatly enhanced.

**Table 4:** Representation of 17 H1N1 T cell epitope sequences among all influenza A 1977–2006 strains and H1N1 strains 2007–2009 that corresponded to the most frequent sequences with at least 88% conservation.

Protein	A/New York/348/2003 H1N1 ELISpot positive peptide§	1977–2006 Influenza A*	2007 human H1N1″	2008 human H1N1̂	2009 human H1N1+
PB1	31 SHGTGTGYTMDTVNRTH 47	99	100	100	100
	120 DKLTQGRQTYDWTLNRN 136	97	100	100	100
	126 RQTYDWTLNRNQPAATA 142	99	100	100	100
	340 APIMFSNKMARLGKGYM 356	96	98	100	92
	-------------R---	2	2	-	8
	489 TFEFTSFFYRYGFVANF 505	100	100	100	100
	495 FFYRYGFVANFSMELPS 511	99	100	100	100
	519 ESADMSIGVTVIKNNMI 535	97	100	100	99
	----------------T	#	-	-	1
	525 IGVTVIKNNMINNDLGP 541	97	100	100	99
	537 NDLGPATAQMALQLFIK 553	98	100	100	99
	S----------------	0.11	-	-	1
	548 LQLFIKDYRYTYRCHRG 564	98	100	100	100
	554 DYRYTYRCHRGDTQIQT 570	98	100	100	99
	------------A----	0.04	-	-	1
PB2	126 KHGTFGPVHFRNQVKIR 142	96	96	-	98
	-Y---------------	#	-	-	1
	---S-------------	#	-	-	1
	-Q---------------	0.14	3	100	-
	132 PVHFRNQVKIRRRVDIN 148	88	100	100	-
	---------------T-	4	-	-	100
	500 RFLRVRDQRGNVLLSPE 516	92	100	100	100
	630 RMQFSSLTVNVRGSGMR 646	97	100	100	-
	---------------L-	1	-	-	100
M1	175 HENRMVLASTTAKAMEQ 191	98	100	100	100
	181 LASTTAKAMEQMAGSSE 197	95	100	100	100

§Highly conserved aa of 1977–2006 influenza A subtypes are boldface.

*3175 PB1, 3144 PB2, and 3781 M1 human H1N1, H3N2, H1N2, H5N1, and avian H5N1 and other avian subtypes sequences circulating between 1977 and 2006 were extracted from NCBI GenBank and GenPept databases as of September 2006. Sequences representing less than 1% were not included unless they were also represented in the 2007–2009 strains.

All human PB1, PB2, and M1 H1N1 sequences from 2007 to 2009 were extracted from the Influenza Virus Resource on Jun 17, 2009.

+168 PB1, 171 PB2, and 280 M1 human H1N1 2009 sequences.

^31 PB1, 31 PB2, and 39 M1 human H1N1 2008 sequences.

″314 PB1, 314 PB2, and 393 M1 human H1N1 2007 sequences.

#New sequence representation not found in the 1977–2006 influenza A subtypes sequences.

**Table 5:** Representation of 9 H1N1 T cell epitope sequences with single amino acid substitutions from the most frequent sequences (≥ 80% conservation) among all influenza A 1977–2006 strains and H1N1 strains 2007–2009.

Protein	A/New York/348/2003 H1N1 ELISpot positive peptide§	1977–2006 Influenza A*	2007 human H1N1″	2008 human H1N1̂	2009 human H1N1+
PB1	---------------K-	86	-	-	99
	37 GYTMDTVNRTHQYSERG 53	13	99	84	-
	-----------R---K-	#	-	-	1
	-----------H-----	#	-	16	-
	----------I------	89	1	-	-
	507 MELPSFGVSGVNESADM 523	10	99	100	100
	----------------L	86	-	-	99
	560 RCHRGDTQIQTRRSFEI 576	11	100	100	-
	------A---------L	0.04	-	-	1
	----S------------	84	-	-	100
	650 GPAKNMEYDAVATTHSW 666	12	99	97	-
	-----I-----------	0.68	-	3	-
	----T------------	0.42	1	-	-
	-----------I-----	87	-	-	96
	656 EYDAVATTHSWVPKRNR 672	11	100	100	-
	-----------T-----	0.76	-	-	4
	-----------K-----	85	-	-	100
	680 RGILEDEQMYQRCCNLF 696	10	98	87	-
	--V--------------	0.23	1	10	-
	----------L------	#	-	3	-
PB2	-------------Q---	89	-	-	100
	434 LLRHFQKDAKVLFLNWG 450	7	97	100	-
	---------R-------	0.03	1	-	-
	----------I------	0.03	1	-	-
	-----------V-----	90	-	-	99
	536 MWEINGPESVLINTYQW 552	8	100	100	1
	-----V-----------	84	-	-	99
	542 PESVLINTYQWIIRNWE 558	8	99	100	1

§Highly conserved aa of 1977–2006 influenza A subtypes are boldface.

*3175 PB1 and 3144 PB2 human H1N1, H3N2, H1N2, H5N1, and avian H5N1 and other avian subtypes sequences circulating between 1977 and 2006 were extracted from NCBI GenBank and GenPept databases as of September 2006. Sequences representing less than 1% were not included unless they were also represented in the 2007–2009 strains.

All human PB1 and PB2 H1N1 sequences from 2007 to 2009 were extracted from the Influenza Virus Resource on Jun 17, 2009.

+168 PB1 and 171 PB2 human H1N1 2009 sequences.

^31 PB1 and 31 PB2 human H1N1 2008 sequences.

″314 PB1 and 314 PB2 human H1N1 2007 sequences.

#New sequence representation not found in the 1977–2006 influenza A subtypes sequences.

## Discussion

An enigma of the immunobiology of influenza A is that vaccines fail to provide long term protection against infection and natural infection does not prevent reinfection. The rapid mutation of the viral proteins, particularly the external HA and NA proteins that are targets for neutralizing antibodies, is credited with a significant role in this loss of immunity. Defective adaptive immunity is also observed with several RNA viruses (including HIV-1 and dengue viruses) with high rates of mutation that result in multiple genetic variants bearing mutated T cell epitope sequences. This has resulted in widespread attention to the use of conserved T cell epitopes of non-structural viral internal proteins [26]–[29]. However, the occurrence of reinfection, despite the human T cell response to conserved sequences after natural infection, suggests the function of a viral mechanism that negatively effects in the host immune response to influenza infection. One possibility is the dual immunosuppressor roles of the influenza A NS1 protein that inhibit innate immunity by preventing type I IFN release, as well as adaptive immunity by attenuating human dendritic cell maturation and the capacity of dendritic cells to induce T cell responses [30]. There is also the concepts of immunological “original sin” and altered epitope ligands where mutations in or adjacent to T cell epitopes preserve binding to MHC molecules but present an altered surface to the original T cell antigen receptor, resulting in an impaired or modified T cell response, including T cell immunosuppression [31]–[37]. Thus, variant viruses bearing mutated T cell epitopes escape memory T cells recognizing the initial epitopes, leading to impaired clearance of variant viruses reinfecting the same individuals.

In this study, HLA transgenic mice, HLA-A2, -A24, -B7, -DR2, -DR3, and DR4, were immunized with 196 overlapping H1N1 peptides of the A/New York/348/2003 strain that contained the highly conserved sequences of the M1, NP, PB1, PB2, and PA proteins of all recorded human and avian influenza A viruses of the past 30 years [12]. Fifty-four (54) of these peptides (22 PB1, 16 PB2, 9 NP, 4 PA, and 3 M1) elicited 63 HLA-restricted T cell responses by IFN-γ ELISpot assay, where 7 peptides were restricted by multiple alleles. It should be recognised that the HLA-restricted T cell activation elicited by these peptides in the transgenic mice identified peptides that contain T cell epitopes, but not the specific identity of the T cell epitopes. While current convention refers to antigenic sequences recognized by T cells in association with an HLA molecule as “epitopes,” this term was introduced by Niels K. Jerne in 1960 in relation to defined structures recognized by antibodies. In contrast, he used the term “cryptotopes” to define the processed peptides that are recognized by T cells in association with an HLA molecule. There are very few sequences called T cell epitopes for which the actual structures of the epitopes are known. Moreover, as is shown in the text, 7 of the peptides elicit responses in 2 or 3 HLA transgenic mice, indicative of multiple or promiscuous epitopes in each peptide.

Many of the epitope peptide sequences reported herein also contain sequences reported by others as T cell epitopes of individuals infected by H1N1, H3N2, and H5N1 viral strains and restricted by a broad range of HLA class I and II alleles. Thus, it is reasonable to expect that the conserved peptides identified here can elicit human T cell epitope responses in the context of several HLA alleles and HLA-supertypes [38] and that the memory T cells can cross-react with epitopes from H1N1, H3N2, and H5N1 [27], [39], [40]. The class I alleles described herein HLA-A*0201, -A*2402, and -B*0702 belong to the distinct supertypes A2, A24, and B7, respectively [10], [11]. HLA class II supertypes are not as well documented but the 3 alleles of the transgenic mice of this study are assigned to supertypes DR1, DR3, and DR4 [41] based on similar protein and three-dimensional structures. Thus, fewer HLA alleles within the supertypes could be selected to protect a broad population in contrast to selection of individual alleles.

Analysis of the conservation and mutational variants of these H1N1 HLA-restricted epitopes was revealing of the marked effect that single aa mutations may have on the representation of T cell epitopes in evolving virus populations. Over the 3 years interval (2007 to 2009) between the database records analyzed by Heiny *et al.* (2006) to the current 2009 H1N1 sequence analysis, only 8 of the 54 highly conserved T cell epitope sequences were without mutational change. These 8 peptides (M1_175–191_, _181–197_, PB1_31–47_, _120–136_, _126–142_, _489–505_, _495–511_, and _548–564_) were representative of almost complete conservation, 95–100%, during the previous recorded history of human H1N1 virus sequences. All others of the identified HLA-restricted T cell epitopes contained at least 1 aa substitution, primarily but not exclusively, of the non-conserved aa of the H1N1 peptides. Our data suggest that the most favorable sequences for a T cell epitope-based vaccine are the 17 H1N1 T cell epitopes of the PB1, PB2, and M1 proteins (Table 4). We are presently investigating the protective effect of these sequences against live influenza challenge. These 17 T cell epitopes were highly conserved over the 33 years (1977–2009) of the examined database records, representing 88 to 100% of all recorded avian and human influenza A viruses, including the H1N1 isolates. Further, they are clustered and have distinct advantages in the design of an epitope-based genetic vaccine, including the retention of native sequences for the function of transporters associated with antigen processing (TAPs) [42] and for the flanking sequences that are reported to modulate epitope processing and function in the selection of immunodominant epitopes [43]. Each of these 17 sequences, except M1_181–197_ and PB1_537–553_, was also characterized by high apparent functional avidity at the lowest peptide concentration of 0.1 µg/ml in the IFN-γ ELISpot assay. Several studies showed that high avidity CD8^+^ T cells were more effective in limiting viral replication *in vitro* [44]–[46]. Further, the 17 T cell epitopes had no identity of 8 or more continuous aa to human peptides that might trigger onset of human autoimmune diseases by priming autoreactive T cells. It is also noteworthy that several of the epitopes are located in described functional domains: PB1_518–575_ in the interacting domain of PB1 with PB2 (PB1_506–659_) [47]; and the overlapping PB2_126–142_ and PB2_132–148_ in the PB1- and NP-binding domain of PB2_1–269_ [48]. T cell epitopes within funtional domains would remain conserved over time as viral mutations useful towards immune escape may disrupt the function of the domains and affect viral fitness unless compensated functionally by multiple co-mutations [49], [50]. Thus, a T cell-based vaccine including these 17 highly conserved T cell epitopes, as described in this study, may possibly greatly reduce, if not eliminate, the incidence of variant amino acids of the corresponding T cell epitopes of any future influenza A pathogen.

## Supporting Information

Figure S1 Selected 196 peptides of Influenza A/New York/348/2003 (H1N1) used for mapping T cell responses in 6 HLA transgenic mouse strains (A2, A24, B7, DR2, DR3, and DR4). Red bold amino acids are conserved residues. Numbers represent residue positions. Overlapping residues were aligned.
(0.05 MB DOC)

Table S1 The first immunization peptide pool consisted of 13 M1 and 84 PB2 peptides of A/New York/348/2003 (H1N1) containing the highly conserved aa.
(0.08 MB DOC)

Table S2 The second immunization peptide pool consisted of 28 NP, 23 PA, and 48 PB1 peptides of A/New York/348/2003 (H1N1) containing the highly conserved aa.
(0.08 MB DOC)

Table S3 Representation of 28 (9 NP, 4 PA, 9 PB2, 5 PB1, and 1 M1) T cell epitope peptides of A/New York/348/2003 (H1N1) among human H1N1, H3N2, H1N2, H5N1, and other avian subtypes circulating between 1977 to 2006.
(0.16 MB DOC)
