# Supplementary material for: Conservation and Diversity of Influenza A H1N1 HLA-Restricted T Cell Epitope Candidates for Epitope-Based Vaccines
Source: PLoS One. 2010 Jan 18;5(1):e8754. doi: 10.1371/journal.pone.0008754 (PMC2807450; doi:10.1371/journal.pone.0008754)
Supplement: Figure S1 — Selected 196 peptides of Influenza A/New York/348/2003 (H1N1) used for mapping T cell responses in 6 HLA transgenic mouse strains (A2, A24, B7, DR2, DR3, and DR4). Red bold amino acids are conserved residues. Numbers represent residue positions. Overlapping residues were aligned. (0.05 MB DOC) [file pone.0008754.s001.doc]

**Figure S1:** Selected 196 peptides of Influenza A/New York/348/2003 (H1N1) used for mapping T cell responses in 6 HLA transgenic mouse strains (A2, A24, B7, DR2, DR3, and DR4). Red bold amino acids are conserved residues. Numbers represent residue positions. Overlapping residues were aligned.

**13 M1 peptides**

1 **MSLLTEVETYVLSI**VPS 17

7 **VETYVLSI**VPSGPLKAE 23

115 IALSYSA**GALASCMGLI** 131

121 A**GALASCMGLIYNRMG**A 137

127 **CMGLIYNRMG**AVTTESA 143

169 TNPLIR**HENRMVLASTT** 185

175 **HENRMVLASTTAKAMEQ** 191

181 **LASTTAKAMEQMAGSSE** 197

187 **KAMEQMAGSSEQAAEAM** 203

193 **AGSSEQAAEAME**VAS**QA** 209

199 **AAEAME**VAS**QARQMVQA** 215

205 VAS**QARQMVQAMRA**IGT 221

210 **RQMVQAMRA**IGTHPSSS 226

**84 PB2 peptides**

1 MERIKELRN**LMSQSRTR** 17

7 LRN**LMSQSRTREILTKT** 23

12 **SQSRTREILTKTTVDHM** 28

18 **EILTKTTVDHMAIIKKY** 34

24 **TVDHMAIIKKYTSGRQE** 40

30 **IIKKYTSGRQEKNP**S**LR** 46

36 **SGRQEKNP**S**LRMKWMMA** 52

42 **NP**S**LRMKWMMAMKYPIT** 58

48 **KWMMAMKYPITADKRI**T 64

54 **KYPITADKRI**TEMI**PER** 70

60 **DKRI**TEMI**PERNEQGQT** 76

66 MI**PERNEQGQTLWSK**VN 82

72 **EQGQTLWSK**VNDAGSDR 88

78 **WSK**VNDAGSDRVMI**SPL** 94

84 AGSDRVMI**SPLAVTWWN** 100

90 MI**SPLAVTWWNRNGP**VA 106

96 **VTWWNRNGP**VANTIHYP 112

102 **NGP**VANTIHYPKIYKTY 118

108 TIHYPKIYKTYFE**KVER** 124

114 IYKTYFE**KVERLKHGTF** 130

120 E**KVERLKHGTFGPVHFR** 136

126 **KHGTFGPVHFRNQVKIR** 142

132 **PVHFRNQVKIRRRVD**IN 148

137 **NQVKIRRRVD**INPGHAD 153

143 **RRVD**INPGHADLSAKEA 159

215 TRFLPVAGGTSSV**YIEV** 231

221 AGGTSSV**YIEVLHLTQG** 237

227 V**YIEVLHLTQGTCWEQM** 243

233 **HLTQGTCWEQMYTPGGE** 249

239 **CWEQMYTPGGE**VR**NDDV** 255

245 **TPGGE**VR**NDDVDQSLII** 261

251 R**NDDVDQSLIIAARNIV** 267

256 **DQSLIIAARNIVRRA**AV 272

262 **AARNIVRRA**AVSADPLA 278

268 **RRA**AVSADPL**ASLLEM** 283

273 SADPL**ASLLEMCHSTQI** 289

279 **SLLEMCHSTQIGG**TRMV 295

285 **HSTQIGG**TRMVDILRQN 301

339 KREEEV**LTGNLQTLK**LT 355

345 **LTGNLQTLK**LT**VHEGYE** 361

351 **TLK**LT**VHEGYEEFTMVG** 367

357 **HEGYEEFTMVG**K**RATAI** 373

363 **FTMVG**K**RATAILRKATR** 379

369 **RATAILRKATRR**LIQLI 385

393 SIVEAIV**VAMVFSQED** 408

398 IV**VAMVFSQEDCM**V**KAV** 414

404 **FSQEDCM**V**KAVRGDLNF** 420

410 **M**V**KAVRGDLNFVNRANQ** 426

416 **GDLNFVNRANQRLNPMH** 432

422 **NRANQRLNPMHQLLRHF** 438

428 **LNPMHQLLRHFQKDAKV** 444

434 **LLRHFQKDAKVLF**LNWG 450

440 **KDAKVLF**LNWGIEHIDN 456

458 MGMIGILP**DMTPSTEMS** 474

464 LP**DMTPSTEMS**MRGV**RV** 480

470 **STEMS**MRGV**RVSKMGVD** 486

476 RGV**RVSKMGVDEYS**NAE 492

482 **KMGVDEYS**NAERVVVSI 498

500 RFLRVRDQR**GNVLLSPE** 516

506 DQR**GNVLLSPEEVSETQ** 522

512 **LLSPEEVSETQG**TEKLT 528

518 **VSETQG**TEK**LTITYSSS** 534

524 TEK**LTITYSSSMMWEIN** 540

530 **TYSSSMMWEINGPESVL** 546

536 **MWEINGPESVL**I**NTYQW** 552

542 **PESVL**I**NTYQWIIRNWE** 558

548 **NTYQWIIRNWE**TVKIQW 564

554 **IRNWE**TVKIQWSQNPTM 570

560 VKIQWSQNPT**MLYNKME** 576

565 SQNPT**MLYNKMEFEPFQ** 581

571 **LYNKMEFEPFQSLVPKA** 587

577 **FEPFQSLVPKA**IRGQYS 593

606 VLGTFDTT**QIIKLLPFA** 622

612 TT**QIIKLLPFAAAPP**K**Q** 628

618 **LLPFAAAPP**K**QSRMQFS** 634

624 **APP**K**QSRMQFSSLTVNV** 640

630 **RMQFSSLTVNVRGSGMR** 646

636 **LTVNVRGSGMRIL**VRGN 652

642 **GSGMRIL**VRGNSPVFNY 658

678 DPDEGTA**GVESAVLRGF** 694

684 A**GVESAVLRGFLI**LGKE 700

690 **VLRGFLIL**GKEDR**RYGP** 706

696 **IL**GKEDR**RYGPALSIN**E 712

**48 PB1 peptides**

1 **MDVNPTLLFLKVP**A**QNA** 17

7 **LLFLKVP**A**QNAISTTFP** 23

13 **P**A**QNAISTTFPYTGDPP** 29

19 **STTFPYTGDPPYSHGTG** 35

25 **TGDPPYSHGTGTGYTMD** 41

31 **SHGTGTGYTMDTVNRTH** 47

37 **GYTMDTVNRTHQYSE**RG 53

43 **VNRTHQYSE**RGRWTKNT 59

108 IETMEV**VQQTRVDKLTQ** 124

114 **VQQTRVDKLTQGRQTYD** 130

120 **DKLTQGRQTYDWTLNRN** 136

126 **RQTYDWTLNRNQPAATA** 142

132 **TLNRNQPAATALANTIE** 148

138 **PAATALANTIE**VFRSNG 154

191 VRDNV**TKKMVTQRTIGK** 207

197 **KKMVTQRTIGKKK**HKLD 213

203 **RTIGKKK**HKLDKRSYLI 219

328 NQPEWFRNI**LSIAPIMF** 344

334 RNI**LSIAPIMFSNKMAR** 350

340 **APIMFSNKMARLGKGYM** 356

346 **NKMARLGKGYMFESK**S**M** 362

352 **GKGYMFESK**S**MKLRTQI** 368

358 **ESK**S**MKLRTQIPAEMLA** 374

364 **LRTQIPAEMLA**NIDLKY 380

465 RFYRTCKLL**GINMSKKK** 481

471 KLL**GINMSKKKSYIN**R**T** 487

477 **MSKKKSYIN**R**TGTFEFT** 493

483 **YIN**R**TGTFEFTSFFYRY** 499

489 **TFEFTSFFYRYGFVANF** 505

495 **FFYRYGFVANFSMELPS** 511

501 **FVANFSMELPSFGVSG**V 517

507 **MELPSFGVSG**V**NESADM** 523

513 **GVSG**V**NESADMSIGVTV** 529

519 **ESADMSIGVTVIKNNMI** 535

525 **IGVTVIKNNMINNDLGP** 541

531 **KNNMINNDLGPATAQMA** 547

537 **NDLGPATAQMALQLFIK** 553

543 **TAQMALQLFIKDYRYTY** 559

548 **LQLFIKDYRYTYRCHRG** 564

554 **DYRYTYRCHRGDTQIQT** 570

560 **RCHRGDTQIQTRRSFE**I 576

566 **TQIQTRRSFEI**KKLWDQ 582

650 GPAKN**MEYDAVATTHSW** 666

656 **EYDAVATTHSW**V**PKRNR** 672

662 **TTHSW**V**PKRNRSILNTS** 678

668 **PKRNRSILNTSQRGILE** 684

674 **ILNTSQRGILEDEQMYQ** 690

680 **RGILEDEQMYQ**RCCNLF 696

**23 PA peptides**

24 YGEDL**KIETNKFAAICT** 40

30 **IETNKFAAICTHLEVCF** 46

36 **AAICTHLEVCFMYSDFH** 52

42 **LEVCFMYSDFHFI**NEQG 58

48 **YSDFHFI**NEQGESIIVE 64

120 IGVTRREVHI**YYLEKAN** 136

126 EVHI**YYLEKANKIKSE**K 142

132 **LEKANKIKSE**K**THIHIF** 148

138 **IKSE**K**THIHIFSFTGEE** 154

144 **HIHIFSFTGEEMA**TKAD 160

150 **FTGEEMA**TKADYTLDEE 166

179 RQEMAS**RGLWDSFRQSE** 195

185 **RGLWDSFRQSERGEETI** 201

191 **FRQSERGEETIEE**RFEI 207

197 **GEETIEE**RFEITGTLRR 213

292 IEDPN**HEGEGIPLYDAI** 308

298 **EGEGIPLYDAIKC**MRTF 314

304 **LYDAIKC**MRTFFGWKEP 320

404 SSWIQN**EFNKACELTDS** 420

410 **EFNKACELTDS**IWIELD 426

552 SAIGQV**SRPMFLYVRTN** 568

558 **SRPMFLYVRTNGTSK**IK 574

564 **YVRTNGTSK**IKMKWGME 580

**28 NP peptides**

1 **MASQGTKRSYEQMET**DG 17

7 **KRSYEQMET**DGERQNAT 23

25 IRASVGRMIG**GIGRFYI** 41

31 RMIG**GIGRFYIQMCTEL** 47

37 **GRFYIQMCTELKL**NDYE 53

43 **MCTELKL**NDYEGRLIQN 59

61 LTIER**MVLSAFDERRN**K 77

67 **VLSAFDERRN**K**YLEEHP** 83

73 **ERRN**K**YLEEHPSAGKDP** 89

79 **LEEHPSAGKDPKKTGGP** 95

85 **AGKDPKKTGGPIY**KRVD 101

91 **KTGGPIY**KRVDGKWVRE 107

103 KWVRELV**LYDKEEIRRI** 119

109 V**LYDKEEIRRIWRQANN** 125

115 **EIRRIWRQANNG**DDATA 131

121 **RQANNG**DDATAGLTHIM 137

127 DDATAGLTHI**MIWHSNL** 143

133 LTHI**MIWHSNLND**TTYQ 149

139 **WHSNLND**TTYQRTRALV 155

234 AQKAMM**DQVRESRNPGN** 250

240 **DQVRESRNPGNAEIEDL** 256

246 **RNPGNAEIEDL**TFLARS 262

402 SAGQIST**QPTFSVQRNL** 418

408 T**QPTFSVQRNLPF**DKTT 424

414 **VQRNLPF**DKTTIMAAFT 430

450 SARPEEVSFQ**GRGVFEL** 466

456 VSFQ**GRGVFELSDE**RAT 472

462 **GVFELSDE**RATNPIVPS 478
