# Supplementary material for: Conservation and Diversity of Influenza A H1N1 HLA-Restricted T Cell Epitope Candidates for Epitope-Based Vaccines
Source: PLoS One. 2010 Jan 18;5(1):e8754. doi: 10.1371/journal.pone.0008754 (PMC2807450; doi:10.1371/journal.pone.0008754)
Supplement: Table S1 — The first immunization peptide pool consisted of 13 M1 and 84 PB2 peptides of A/New York/348/2003 (H1N1) containing the highly conserved aa. (0.08 MB DOC) [file pone.0008754.s002.doc]

Table S1: The first immunization peptide pool consisted of 13 M1 and 84 PB2 peptides of A/New York/348/2003 (H1N1) containing the highly conserved aa (boldface).

| **Protein** | **Peptides** | **Protein** | **Sequences** |
| --- | --- | --- | --- |
| **M1** | 1 **MSLLTEVETYVLSI**VPS 17 | **PB2** | 279 **SLLEMCHSTQIGG**TRMV 295 |
|  | 7 **VETYVLSI**VPSGPLKAE 23 |  | 285 **HSTQIGG**TRMVDILRQN 301 |
|  | 115 IALSYSA**GALASCMGLI** 131 |  | 339 KREEEV**LTGNLQTLK**LT 355 |
|  | 121 A**GALASCMGLIYNRMG**A 137 |  | 345 **LTGNLQTLK**LT**VHEGYE** 361 |
|  | 127 **CMGLIYNRMG**AVTTESA 143 |  | 351 **TLK**LT**VHEGYEEFTMVG** 367 |
|  | 169 TNPLIR**HENRMVLASTT** 185 |  | 357 **HEGYEEFTMVG**K**RATAI** 373 |
|  | 175 **HENRMVLASTTAKAMEQ** 191 |  | 363 **FTMVG**K**RATAILRKATR** 379 |
|  | 181 **LASTTAKAMEQMAGSSE** 197 |  | 369 **RATAILRKATRR**LIQLI 385 |
|  | 187 **KAMEQMAGSSEQAAEAM** 203 |  | 393 SIVEAIV**VAMVFSQED** 408 |
|  | 193 **AGSSEQAAEAME**VAS**QA** 209 |  | 398 IV**VAMVFSQEDCM**V**KAV** 414 |
|  | 199 **AAEAME**VAS**QARQMVQA** 215 |  | 404 **FSQEDCM**V**KAVRGDLNF** 420 |
|  | 205 VAS**QARQMVQAMRA**IGT 221 |  | 410 **M**V**KAVRGDLNFVNRANQ** 426 |
|  | 210 **RQMVQAMRA**IGTHPSSS 226 |  | 416 **GDLNFVNRANQRLNPMH** 432 |
| **PB2** | 1 MERIKELRN**LMSQSRTR** 17 |  | 422 **NRANQRLNPMHQLLRHF** 438 |
|  | 7 LRN**LMSQSRTREILTKT** 23 |  | 428 **LNPMHQLLRHFQKDAKV** 444 |
|  | 12 **SQSRTREILTKTTVDHM** 28 |  | 434 **LLRHFQKDAKVLF**LNWG 450 |
|  | 18 **EILTKTTVDHMAIIKKY** 34 |  | 440 **KDAKVLF**LNWGIEHIDN 456 |
|  | 24 **TVDHMAIIKKYTSGRQE** 40 |  | 458 MGMIGILP**DMTPSTEMS** 474 |
|  | 30 **IIKKYTSGRQEKNP**S**LR** 46 |  | 464 LP**DMTPSTEMS**MRGVRV 480 |
|  | 36 **SGRQEKNP**S**LRMKWMMA** 52 |  | 470 **STEMS**MRGV**RVSKMGVD** 486 |
|  | 42 **NP**S**LRMKWMMAMKYPIT** 58 |  | 476 RGV**RVSKMGVDEYS**NAE 492 |
|  | 48 **KWMMAMKYPITADKRI**T 64 |  | 482 **KMGVDEYS**NAERVVVSI 498 |
|  | 54 **KYPITADKRI**TEMI**PER** 70 |  | 500 RFLRVRDQR**GNVLLSPE** 516 |
|  | 60 **DKRI**TEMI**PERNEQGQT** 76 |  | 506 DQR**GNVLLSPEEVSETQ** 522 |
|  | 66 MI**PERNEQGQTLWSK**VN 82 |  | 512 **LLSPEEVSETQG**TEKLT 528 |
|  | 72 **EQGQTLWSK**VNDAGSDR 88 |  | 518 **VSETQG**TEK**LTITYSSS** 534 |
|  | 78 **WSK**VNDAGSDRVMI**SPL** 94 |  | 524 TEK**LTITYSSSMMWEIN** 540 |
|  | 84 AGSDRVMI**SPLAVTWWN** 100 |  | 530 **TYSSSMMWEINGPESVL** 546 |
|  | 90 MI**SPLAVTWWNRNGP**VA 106 |  | 536 **MWEINGPESVL**I**NTYQW** 552 |
|  | 96 **VTWWNRNGP**VANTIHYP 112 |  | 542 **PESVL**I**NTYQWIIRNWE** 558 |
|  | 102 **NGP**VANTIHYPKIYKTY 118 |  | 548 **NTYQWIIRNWE**TVKIQW 564 |
|  | 108 TIHYPKIYKTYFE**KVER** 124 |  | 554 **IRNWE**TVKIQWSQNPTM 570 |
|  | 114 IYKTYFE**KVERLKHGTF** 130 |  | 560 VKIQWSQNPT**MLYNKME** 576 |
|  | 120 E**KVERLKHGTFGPVHFR** 136 |  | 565 SQNPT**MLYNKMEFEPFQ** 581 |
|  | 126 **KHGTFGPVHFRNQVKIR** 142 |  | 571 **LYNKMEFEPFQSLVPKA** 587 |
|  | 132 **PVHFRNQVKIRRRVD**IN 148 |  | 577 **FEPFQSLVPKA**IRGQYS 593 |
|  | 137 **NQVKIRRRVD**INPGHAD 153 |  | 606 VLGTFDTT**QIIKLLPFA** 622 |
|  | 143 **RRVD**INPGHADLSAKEA 159 |  | 612 TT**QIIKLLPFAAAPP**K**Q** 628 |
|  | 215 TRFLPVAGGTSSV**YIEV** 231 |  | 618 **LLPFAAAPP**K**QSRMQFS** 634 |
|  | 221 AGGTSSV**YIEVLHLTQG** 237 |  | 624 **APP**K**QSRMQFSSLTVNV** 640 |
|  | 227 V**YIEVLHLTQGTCWEQM** 243 |  | 630 **RMQFSSLTVNVRGSGMR** 646 |
|  | 233 **HLTQGTCWEQMYTPGGE** 249 |  | 636 **LTVNVRGSGMRIL**VRGN 652 |
|  | 239 **CWEQMYTPGGEV**R**NDDV** 255 |  | 642 **GSGMRIL**VRGNSPVFNY 658 |
|  | 245 **TPGGEV**R**NDDVDQSLII** 261 |  | 678 DPDEGTA**GVESAVLRGF** 694 |
|  | 251 R**NDDVDQSLIIAARNIV** 267 |  | 684 A**GVESAVLRGFLI**LGKE 700 |
|  | 256 **DQSLIIAARNIVRRA**AV 272 |  | 690 **VLRGFLI**LGKEDR**RYGP** 706 |
|  | 262 **AARNIVRRA**AVSADPL**A** 278 |  | 696 **I**LGKEDR**RYGPALSIN**E 712 |
|  | 268 **RRA**AVSADPL**ASLLEM** 283 |  | 702 R**RYGPALSIN**ELSNLAK 718 |
|  | 273 SADPL**ASLLEMCHSTQI** 289 |  |  |
