# Supplementary material for: Conservation and Diversity of Influenza A H1N1 HLA-Restricted T Cell Epitope Candidates for Epitope-Based Vaccines
Source: PLoS One. 2010 Jan 18;5(1):e8754. doi: 10.1371/journal.pone.0008754 (PMC2807450; doi:10.1371/journal.pone.0008754)
Supplement: Table S2 — The second immunization peptide pool consisted of 28 NP, 23 PA, and 48 PB1 peptides of A/New York/348/2003 (H1N1) containing the highly conserved aa. (0.08 MB DOC) [file pone.0008754.s003.doc]

**Table S2: The second immunization peptide pool consisted of 28 NP, 23 PA, and 48 PB1 peptides of A/New York/348/2003 (H1N1) containing the highly conserved aa (boldface).**

| **Protein** | **Sequences** | **Protein** | **Sequences** |
| --- | --- | --- | --- |
| **NP** | 1 **MASQGTKRSYEQMET**DG 17 | **PB1** | 1 **MDVNPTLLFLKVP**A**QNA** 17 |
|  | 7 **KRSYEQMET**DGERQNAT 23 |  | 7 **LLFLKVP**A**QNAISTTFP** 23 |
|  | 25 IRASVGRMIG**GIGRFYI** 41 |  | 13 **P**A**QNAISTTFPYTGDPP** 29 |
|  | 31 RMIG**GIGRFYIQMCTEL** 47 |  | 19 **STTFPYTGDPPYSHGTG** 35 |
|  | 37 **GRFYIQMCTELKL**NDYE 53 |  | 25 **TGDPPYSHGTGTGYTMD** 41 |
|  | 43 **MCTELKL**NDYEGRLIQN 59 |  | 31 **SHGTGTGYTMDTVNRTH** 47 |
|  | 61 LTIER**MVLSAFDERRN**K 77 |  | 37 **GYTMDTVNRTHQYSE**RG 53 |
|  | 67 **VLSAFDERRN**K**YLEEHP** 83 |  | 43 **VNRTHQYSE**RGRWTKNT 59 |
|  | 73 **ERRN**K**YLEEHPSAGKDP** 89 |  | 108 IETMEV**VQQTRVDKLTQ** 124 |
|  | 79 **LEEHPSAGKDPKKTGGP** 95 |  | 114 **VQQTRVDKLTQGRQTYD** 130 |
|  | 85 **AGKDPKKTGGPIY**KRVD 101 |  | 120 **DKLTQGRQTYDWTLNRN** 136 |
|  | 91 **KTGGPIY**KRVDGKWVRE 107 |  | 126 **RQTYDWTLNRNQPAATA** 142 |
|  | 103 KWVRELV**LYDKEEIRRI** 119 |  | 132 **TLNRNQPAATALANTIE** 148 |
|  | 109 V**LYDKEEIRRIWRQANN** 125 |  | 138 **PAATALANTIE**VFRSNG 154 |
|  | 115 **EIRRIWRQANNG**DDATA 131 |  | 191 VRDNV**TKKMVTQRTIGK** 207 |
|  | 121 **RQANNG**DDATAGLTHI**M** 137 |  | 197 **KKMVTQRTIGKKK**HKLD 213 |
|  | 127 DDATAGLTHI**MIWHSNL** 143 |  | 203 **RTIGKKK**HKLDKRSYLI 219 |
|  | 133 LTHI**MIWHSNLND**TTYQ 149 |  | 328 NQPEWFRNI**LSIAPIMF** 344 |
|  | 139 **WHSNLND**TTYQRTRALV 155 |  | 334 RNI**LSIAPIMFSNKMAR** 350 |
|  | 234 AQKAMM**DQVRESRNPGN** 250 |  | 340 **APIMFSNKMARLGKGYM** 356 |
|  | 240 **DQVRESRNPGNAEIEDL** 256 |  | 346 **NKMARLGKGYMFESK**S**M** 362 |
|  | 246 **RNPGNAEIEDL**TFLARS 262 |  | 352 **GKGYMFESK**S**MKLRTQI** 368 |
|  | 402 SAGQIST**QPTFSVQRNL** 418 |  | 358 **ESK**S**MKLRTQIPAEMLA** 374 |
|  | 408 T**QPTFSVQRNLPF**DKTT 424 |  | 364 **LRTQIPAEMLA**NIDLKY 380 |
|  | 414 **VQRNLPF**DKTTIMAAFT 430 |  | 465 RFYRTCKLL**GINMSKKK** 481 |
|  | 450 SARPEEVSFQ**GRGVFEL** 466 |  | 471 KLL**GINMSKKKSYIN**R**T** 487 |
|  | 456 VSFQ**GRGVFELSDE**RAT 472 |  | 477 **MSKKKSYIN**R**TGTFEFT** 493 |
|  | 462 **GVFELSDE**RATNPIVPS 478 |  | 483 **YIN**R**TGTFEFTSFFYRY** 499 |
| **PA** | 24 YGEDL**KIETNKFAAICT** 40 |  | 489 **TFEFTSFFYRYGFVANF** 505 |
|  | 30 **IETNKFAAICTHLEVCF** 46 |  | 495 **FFYRYGFVANFSMELPS** 511 |
|  | 36 **AAICTHLEVCFMYSDFH** 52 |  | 501 **FVANFSMELPSFGVSG**V 517 |
|  | 42 **LEVCFMYSDFHFI**NEQG 58 |  | 507 **MELPSFGVSG**V**NESADM** 523 |
|  | 48 **YSDFHFI**NEQGESIIVE 64 |  | 513 **GVSG**V**NESADMSIGVTV** 529 |
|  | 120 IGVTRREVHI**YYLEKAN** 136 |  | 519 **ESADMSIGVTVIKNNMI** 535 |
|  | 126 EVHI**YYLEKANKIKSE**K 142 |  | 525 **IGVTVIKNNMINNDLGP** 541 |
|  | 132 **LEKANKIKSE**K**THIHIF** 148 |  | 531 **KNNMINNDLGPATAQMA** 547 |
|  | 138 **IKSE**K**THIHIFSFTGEE** 154 |  | 537 **NDLGPATAQMALQLFIK** 553 |
|  | 144 **HIHIFSFTGEEMA**TKAD 160 |  | 543 **TAQMALQLFIKDYRYTY** 559 |
|  | 150 **FTGEEMA**TKADYTLDEE 166 |  | 548 **LQLFIKDYRYTYRCHRG** 564 |
|  | 179 RQEMAS**RGLWDSFRQSE** 195 |  | 554 **DYRYTYRCHRGDTQIQT** 570 |
|  | 185 **RGLWDSFRQSERGEETI** 201 |  | 560 **RCHRGDTQIQTRRSFE**I 576 |
|  | 191 **FRQSERGEETIEE**RFEI 207 |  | 566 **TQIQTRRSFE**IKKLWDQ 582 |
|  | 197 **GEETIEE**RFEITGTLRR 213 |  | 650 GPAKN**MEYDAVATTHSW** 666 |
|  | 292 IEDPN**HEGEGIPLYDAI** 308 |  | 656 **EYDAVATTHSW**V**PKRNR** 672 |
|  | 298 **EGEGIPLYDAIKC**MRTF 314 |  | 662 **TTHSW**V**PKRNRSILNTS** 678 |
|  | 304 **LYDAIKC**MRTFFGWKEP 320 |  | 668 **PKRNRSILNTSQRGILE** 684 |
|  | 404 SSWIQN**EFNKACELTDS** 420 |  | 674 **ILNTSQRGILEDEQMYQ** 690 |
|  | 410 **EFNKACELTDS**IWIELD 426 |  | 680 **RGILEDEQMYQ**RCCNLF 696 |
|  | 552 SAIGQV**SRPMFLYVRTN** 568 |  |  |
|  | 558 **SRPMFLYVRTNGTSK**IK 574 |  |  |
|  | 564 **YVRTNGTSK**IKMKWGME 580 |  |  |
