# Supplementary material for: Conservation and Diversity of Influenza A H1N1 HLA-Restricted T Cell Epitope Candidates for Epitope-Based Vaccines
Source: PLoS One. 2010 Jan 18;5(1):e8754. doi: 10.1371/journal.pone.0008754 (PMC2807450; doi:10.1371/journal.pone.0008754)
Supplement: Table S3 — Representation of 28 (9 NP, 4 PA, 9 PB2, 5 PB1, and 1 M1) T cell epitope peptides of A/New York/348/2003 (H1N1) among human H1N1, H3N2, H1N2, H5N1, and other avian subtypes circulating between 1977 to 2006. (0.16 MB DOC) [file pone.0008754.s004.doc]

Table S3: Representation of 28 (9 NP, 4 PA, 9 PB2, 5 PB1, and 1 M1) T cell epitope peptides of A/New York/348/2003 (H1N1) among human H1N1, H3N2, H1N2, H5N1, and other avian subtypes circulating between 1977 to 2006.

| Protein | A/New York/348/2003 H1N1 ELISpot positive peptide**§** | 1977-2006 influenza A* |
| --- | --- | --- |
| NP | **---------**G------- | 39 |
|  | **---------**--D----- | 31 |
|  | 7 **KRSYEQMET**DGERQNAT 23 | 22 |
|  | ---------G----D—- | 3 |
|  | ---------S-----—- | 1 |
|  | K—D-----------—- | 42 |
|  | --V------------—- | 28 |
|  | --VS-----------—- | 11 |
|  | 31 RMIG**GIGRFYIQMCTEL** 47 | 8 |
|  | --V-------V----—- | 3 |
|  | K--------------—- | 2 |
|  | ---D-----------—- | 2 |
|  | ---S-----------—- | 2 |
|  | -------------S--- | 75 |
|  | -------------S-H- | 9 |
|  | 37 **GRFYIQMCTELKL**NDYE 53 | 8 |
|  | -------------S-Q- | 1 |
|  | ----V--------S--- | 1 |
|  | -----------Q-S--- | 1 |
|  | ----R------------ | 49 |
|  | 73 **ERRN**K**YLEEHPSAGKDP** 89 | 45 |
|  | ----R----N------- | 2 |
|  | ------I---------- | 24 |
|  | --M-------------- | 22 |
|  | R-M-------------- | 21 |
|  | --M---I---------- | 16 |
|  | 103 KWVRELV**LYDKEEIRRI** 119 | 7 |
|  | --M---I---------V | 3 |
|  | --I---I---------- | 2 |
|  | --M---I----D----- | 1 |
|  | 109 V**LYDKEEIRRIWRQANN** 125 | 50 |
|  | I---------------- | 41 |
|  | I---------V------ | 3 |
|  | I----D----------- | 1 |
|  | ---L---------A--- | 38 |
|  | ---M------------- | 25 |
|  | ---M---------A--- | 17 |
|  | -------------A--- | 12 |
|  | 133 LTHI**MIWHSNLND**TTYQ 149 | 7 |
|  | ------V---------- | 69 |
|  | ----T-V---------- | 10 |
|  | 402 SAGQIST**QPTFSVQRNL** 418 | 6 |
|  | ------I---------- | 5 |
|  | ------V—A-------- | 5 |
|  | ------V--------S- | 3 |
|  | V------------E-S- | 41 |
|  | V------------ERA- | 35 |
|  | 408 T**QPTFSVQRNLPF**DKTT 424 | 6 |
|  | I---------------- | 3 |
|  | V--------S---ERA- | 3 |
|  | V—A-----------P-- | 2 |
|  | V------------ERS- | 1 |
| PA | 42 **LEVCFMYSDFHFI**NEQG 58 | 58 |
|  | -------------D-R- | 27 |
|  | -------------D-RS | 9 |
|  | -------------D--- | 1 |
|  | ---------------R- | 1 |
|  | --I----------D-R- | 1 |
|  | ---------------L- | 1 |
|  | ----------------N | 47 |
|  | 126 EVHI**YYLEKANKIKSE**K 142 | 37 |
|  | ---T------------- | 9 |
|  | ----------------R | 1 |
|  | -I--------------- | 1 |
|  | ----------------E | 1 |
|  | ---------S------- | 1 |
|  | ----------N------ | 47 |
|  | 132 **LEKANKIKSE**K**THIHIF** 148 | 47 |
|  | ----------R------ | 2 |
|  | ----------E------ | 1 |
|  | ---S------------- | 1 |
|  | 558 **SRPMFLYVRTNGTSK**IK 574 | 65 |
|  | ---------------V- | 32 |
| PB2 | 42 **NP**S**LRMKWMMAMKYPIT** 58 | 60 |
|  | --A-------------- | 39 |
|  | 48 **KWMMAMKYPITADKRI**T 64 | 57 |
|  | ----------------M | 28 |
|  | ----------------I | 8 |
|  | --------------K-- | 2 |
|  | -------------V--- | 47 |
|  | ----------M------ | 25 |
|  | 54 **KYPITADKRI**TEMI**PER** 70 | 9 |
|  | ----------I------ | 7 |
|  | --------K-------- | 2 |
|  | ----------MD----- | 1 |
|  | 256 **DQSLIIAARNIVRRA**AV 272 | 61 |
|  | ---------------T- | 34 |
|  | ----V------------ | 2 |
|  | ---------------I- | 1 |
|  | -------------V--- | 47 |
|  | 369 **RATAILRKATRR**LIQLI 385 | 46 |
|  | ------------MI--- | 3 |
|  | 458 MGMIGILP**DMTPSTEMS** 474 | 43 |
|  | ---V-V----------- | 39 |
|  | -----V----------- | 5 |
|  | ---V------------- | 4 |
|  | -------S--------- | 1 |
|  | --------------I-- | 46 |
|  | -----------L----- | 25 |
|  | -----------L--I-- | 10 |
|  | 464 LP**DMTPSTEMS**MRGVRV 480 | 10 |
|  | 524 TEK**LTITYSSSMMWEIN** 540 | 46 |
|  | --R-------------- | 46 |
|  | M---------------- | 3 |
|  | I-R-------------- | 1 |
|  | 548 **NTYQWIIRNWE**TVKIQW 564 | 54 |
|  | -----------A----- | 35 |
|  | -----V----------- | 6 |
|  | -----------I----- | 1 |
| PB1 | 328 NQPEWFRNI**LSIAPIMF** 344 | 55 |
|  | --------V-------- | 39 |
|  | K-------V-------- | 1 |
|  | -----------M----- | 1 |
|  | 352 **GKGYMFESK**S**MKLRTQI** 368 | 47 |
|  | ---------R------- | 47 |
|  | -R--------------- | 2 |
|  | ----------------V | 1 |
|  | ---------N------- | 1 |
|  | --------R-------- | 1 |
|  | 358 **ESK**S**MKLRTQIPAEMLA** 374 | 47 |
|  | ---R------------- | 46 |
|  | ----------V------ | 1 |
|  | --R-------------- | 1 |
|  | --------V-------- | 75 |
|  | ----I---V-------- | 13 |
|  | 465 RFYRTCKLL**GINMSKKK** 481 | 10 |
|  | --V------------K- | 46 |
|  | --V-------------- | 43 |
|  | 471 KLL**GINMSKKKSYIN**R**T** 487 | 10 |
| M1 | 169 TNPLIR**HENRMVLASTT** 185 | 72 |
|  | -----K----------- | 25 |
|  | ------------I---- | 1 |

§ Highly conserved aa are boldface.

* 3175 PB1, 3144 PB2, and 3781 M1 human H1N1, H3N2, H1N2, H5N1, and avian H5N1 and other avian subtypes sequences circulating between 1977 and 2006 were extracted from NCBI GenBank and GenPept databases as of September 2006. Sequences representing less than 1% of each dataset were excluded.
